# Supplementary material for: Parasitic contamination of fresh vegetables and fruits sold in open-air markets in peri-urban areas of Jimma City, Oromia, Ethiopia: A community-based cross-sectional study
Source: PLoS One. 2024 Mar 21;19(3):e0290655. doi: 10.1371/journal.pone.0290655 (PMC10956833; doi:10.1371/journal.pone.0290655)
Supplement: S1 Table — (DOCX) [file pone.0290655.s002.docx]

**S1** **Table:** Socio-demographic and characteristics of the study participants in peri-urban areas of Jimma city from July-September, 2021.

| Variables | Category | Frequency | Percentage |
| --- | --- | --- | --- |
| Gender of sellers | Male | 33 | 8.8 |
|  | Female | 342 | 91.2 |
| Age of sellers | <19 years | 5 | 1.3 |
|  | 20-29 years | 222 | 59.2 |
|  | 30-39 years | 134 | 35.7 |
|  | 40 and above years | 14 | 3.7 |
| Educational status | No formal education | 58 | 15.5 |
|  | Primary education | 181 | 48.3 |
|  | Secondary education | 130 | 34.7 |
|  | Higher education | 6 | 1.6 |
| Marital status | Single | 83 | 22.1 |
|  | Married | 262 | 69.9 |
|  | Divorced | 19 | 5.1 |
|  | Widowed | 11 | 2.9 |
| Average monthly income | <1500ETB | 60 | 16.0 |
|  | 1500-2000ETB | 177 | 47.2 |
|  | 2000-2500ETB | 123 | 32.8 |
|  | 2500-3000ETB | 14 | 3.7 |
|  | >3000ETB | 1 | 0.3 |
| Experience of sellers | <1 | 21 | 5.6 |
|  | 1-2 | 89 | 23.7 |
|  | 3-5 | 184 | 49.1 |
|  | >5 | 81 | 21.6 |
| Market sites of sample collection | Bore | 143 | 38.1 |
|  | Hora Gibie | 100 | 26.7 |
|  | Jiren | 132 | 35.2 |
| Source of vegetable | Farmers | 212 | 56.5 |
|  | Middle Men | 153 | 40.8 |
|  | Private Garden | 10 | 2.7 |
| Sellers’ hygiene condition | Adequate | 118 | 31.5 |
|  | Inadequate | 257 | 68.5 |
| Cleanness of the containers | Adequate | 101 | 26.9 |
|  | Inadequate | 274 | 73.1 |
| Wash status | Yes | 160 | 42.7 |
|  | No | 215 | 57.3 |
| Source of water | Pipe | 24 | 3.75 |
|  | Well | 99 | 61.9 |
|  | River | 37 | 3.74 |
| Means of display | Floor | 288 | 76.8 |
|  | Shelf | 57 | 15.2 |
|  | Table | 30 | 8.0 |
