# Supplementary material for: Parasitic contamination of fresh vegetables and fruits sold in open-air markets in peri-urban areas of Jimma City, Oromia, Ethiopia: A community-based cross-sectional study
Source: PLoS One. 2024 Mar 21;19(3):e0290655. doi: 10.1371/journal.pone.0290655 (PMC10956833; doi:10.1371/journal.pone.0290655)
Supplement: Attachment — Submitted filename: Response to Reviewers.docx [file pone.0290655.s003.docx]

**Date: May 05, 2023**

**To: Adriana Calderaro, Academic Editor**

**PLOS ONE**

**Issue:** Submission of revised manuscript - PONE-D-23-01852R1

Thank you for giving me the opportunity to submit a revised version of our manuscript entitled **“*Parasitic contamination of fresh vegetables and fruits sold in open-air markets in peri-urban areas of Jimma City, Oromia, Ethiopia: A community-based cross-sectional study*”** to PLOS ONE.

We appreciate the time and effort that editor and the reviewers have dedicated to providing your valuable feedback on our manuscript. We are so grateful to you for your insightful comments. We have been able to incorporate changes to reflect suggestion provided. We have highlighted the changes within the revised version of the manuscript.

Here is a point-by-point response to the reviewers’ comments and concerns;

**Reviewer #1:**

“This study has been well carried out and shows how much dirty vegetables and fruits could transmit parasitic infections to humans. The experimentally procedure and statistically analysis is done with classical methods and the English grammatical form is corrected and well understandable. The only note to Authors is: how do you can distinguish Giardia cysts at specific level with only the microscopical assay? It could be better to indicate this parasite presence with Giardia sp. definition. Please, note also that Cryptosporidium is incorrectly written (Cyreptosporidium) and that spp. does not require capital letter (Spp), in both Figure 2 and 3.”

We would like to thank the reviewer#1 for his constructive feedback. The manuscript was improved with the requested recommendations and each comment has been addressed as following:

**Question 1:** How do you can distinguish Giardia cysts at specific level with only the microscopical assay? It could be better to indicate this parasite presence with Giardia spp. definition.

**Response:** Modified as suggested by the reviewer in the revised version of the manuscript. Thank you!

**Comment 2:** Please, note also that Cryptosporidium is incorrectly written (Cyreptosporidium) and that spp. does not require capital letter (Spp), in both Figure 2 and 3.

**Response:** We fully agree with this comment and to address it, the following change was done:

- **“**Cyreptosporidium” replaced with “Cryptosporidium”
- “Spp” replaced with “spp.”

**Reviewer #2:**

We would like to thank Reviewer #2 for his comments which contributed to improve the manuscript. The manuscript was improved with the requested recommendations and each comment has been addressed. Additional details and discussion was added to clarify and compare our data. Each of the comments was addressed as following:

**Major comments:
Comment 1:** All rounding to decimal places must be checked as there are errors in all tables.
**Response:** All rounding to decimal places are **thoroughly** checked and errors are corrected in the revised version of the manuscript. Thank you!

**Comment 2:** The abbreviation “spp.” must always be written in lower case and not italics. Do the respective changes in all text and tables.
**Response**: Corrected throughout the entire text and tables in the revised version of the manuscript. Thank you!

**Comment 3:** Authors should always consider using the same designation for Giardia, lamblia or intestinalis (only one name), throughout the entire text.

**Response:** This comment was also done by Reviewer 1 and to address it, the designations for “Giardia, lamblia or intestinalis” throughout the entire text are modified with “Giardia spp.” by considering the difficulty microscopical assay for identification of the detected Giardia cysts in this study.

**Comment 4:** The authors do not explain whether the collection of samples was made in the rainy season or in the dry season, or summer or winter. The fact that they say that the collection was made from July to September does not indicate, for the reader, the degree of rainfall and temperature.
**Response:** We fully agree with this comment and to address it, the sample collection season is added as “during major rainy season” (Line 117). Degree of rain fall and temperature is also included (Lines 121-122).

**Comment 5:** In the introduction, results and discussion, the authors did not take into account a similar study that was carried out in Mozambique and published in 2021, involving similar populations. This study should be introduced in this article and compared with the results you obtained because it is a recent study, carried out in East Africa.

“Salamandane et al.,. (2021). Occurrence of intestinal parasites of public health significance in fresh horticultural products sold in Maputo markets and supermarkets, Mozambique. Microorganisms, 9, 1806. <https://doi.org/10.3390/microorganisms9091806>”

**Response:** To address this comment the following information from requested references was added in the introduction and discussion sections:

“There are plenty of open markets where fruits and vegetables are sold in Africa. Fresh produce is exposed to the environment in these markets, including domestic animals, which add to the risk of food contamination [6, 9, 10, 11]. On this continent, studies on the prevalence of intestinal parasites associated with vegetables and fruits have been performed mostly in Mozambique, and Ghana [9, 10, 11].”**(Lines 97-101)**

**Minor comments:
Background:**
Line 105 – “vegetables and fruits….”
**Response**: Corrected (Line 110)
**Parasitological examination:**
Line 145, 147 – “modified Ziehl–Neelsen….”

**Response**: Corrected (Line 150, 152)
Line 145 – Since Cryptosporidium is not a coccidia, authors should write “detection of coccidian and Cryptosporidium oocysts….”
**Response**: Corrected (Line 150-151)
**Results:**
Line 162 – “37.0%” instead of “37.3%”

**Response**: Corrected; “37.3%” replaced with “37 %”( Line 176). Thank you!

Line 172 – “ Cryptosporidium spp.”
**Response**: Corrected (Line 185)
Lines 173-174 – “S. stercoralis”
**Response**: Corrected (Line 186)
Line 175 – “Cryptosporidium (9.2%) oocysts”
**Response**: Corrected (Line 188)
Line 175 – which forms of development were identified for Toxocara and Fasciola?
**Response:** the identified parasitic stages for both Toxocara and Fasciola parasites were **eggs**. The detected forms of development was depicted in the revised version of the manuscript (Line188)

Lines 181-182 – “detected in carrot 63.8% (30), followed by lettuce 63.1% (29) and Cabbage 61.7% (29). Banana with a prevalence of 14.9% (7)…”
**Response**: Corrected (Line 193-194)
Line 183 – “A. lumbricodes was abundantly…”
**Response**: Corrected (Line 195)
**Discussion:**
Lines 211-214 – The names of vegetables and fruits should be in low case “lettuce….”
**Response**: Corrected (Line 223-226)
Line 221 – Authors should discuss the difference of contamination rate found in their study and in the study done also in Kenya (reference 15).
**Response**: To address this comment the following information was added:

“Our finding is explained by the fact that such markets have more frequent running water during the main rainy season, which increases the likelihood that producers and vendors will wash their products thoroughly. Another hypothesis is assuming that parasite forms on the surface of vegetables are washed away by rain [9]. Moreover, since 2015, water, sanitation, and hygiene (WASH) activities, health education, and biannual deworming programs for helminthes have been conducted and substantially decreased environmental fecal contamination as well as parasitic contamination in Ethiopia [6]. The difference may also be due to variations in the types and quantities of fruits and vegetables evaluated.” **(Lines 231-240)**

Lines 228-237 - In this paragraph the authors don’t explain the differences between the three markets analyzed to try to justify the differences of contamination rates.

**Response**: the differences of contamination rates among the three markets analyzed are depicted as “The differences between the three markets might be due to the different sources of vegetables as well as the hygienic practices in handling and washing by different sellers. Moreover, the high prevalence of intestinal parasites on vegetables is due to the fact that these open-air markets were characterized by poor sewage disposal systems, and vegetables were brought in from rural areas around these markets.” (246-250)

Lines 240-241 – “Banana was the least contaminated fruit (14.9%).”

**Response**: Corrected (Line 253)

Line 248 – “A. lumbricoides”.

**Response**: Corrected (Line 263)

Line 249 – “was” instead of “were”.

**Response**: Corrected (Line 263)

Line 258 and 260 – “S. stercoralis”.

Response: Corrected (Line 271 & 273)

Line 261 – “Strongyloides spp.”.

**Response**: Corrected (Line 274)

Line 264 – In the begin of a paragrapgh don’t use an abbreviation of a name “Entamoeba histolytica/dispar cysts”.

**Response**: Corrected (Line 277)

Line 266 – Authors refer to two articles “references 10 and 13” but don’t explain what was the deference of findings.

**Response**: the paragraph rephrased as the following “However, the rate of contamination was lower as compared to a study finding from Arba Minch, Ethiopia [13]. Variation in the prevalence of *E. histolytica/E. dispar* and *Giardia* spp*.* might be attributed to the long periods of survival of the cysts under cool and moist conditions and variations in geographical distribution [6].” (Line 279-280)

Line 270 – Beginning of paragraph “Hymenolepis nana”.

**Response**: Corrected (Line 283)

Lines 275, 278, 283, 285 – “Cryptosporidium”.

Response: Corrected (Line 288, 296, 301, 304)

Lines 275-279 - the authors compare the results they obtained with the results of other studies, but do not indicate what these other results were.

Response: Corrected (Line 288-296)

Line 286, 289 – “Toxocara spp.”

Response: Corrected (Lines 305, 308)

Line 291 – “Toxocara eggs”

Response: Corrected (Line 310)

Line 293 – “Fasciola”

Response: Corrected (Line 312)

Lines 295-296 – Authors write is not a “Some Fasciola spp are known to be neglected zoonotic diseases [25]”. Fasciola is a parasite that causes disease. Consider revision the phrase.

**Response**: We rephrased the sentence as requested by the reviewer (Line 314-315)

Lines 297-304 – In this area, the results that you obtained have to be compared with results of other studies, namely the article Salamandane et al, 2021.

Response: Corrected (Line 323-327)
