# Supplementary material for: Parasitic contamination of fresh vegetables and fruits sold in open-air markets in peri-urban areas of Jimma City, Oromia, Ethiopia: A community-based cross-sectional study
Source: PLoS One. 2024 Mar 21;19(3):e0290655. doi: 10.1371/journal.pone.0290655 (PMC10956833; doi:10.1371/journal.pone.0290655)
Supplement: Attachment — Submitted filename: Response to Reviewers.docx [file pone.0290655.s004.docx]

**Date: July 26, 2023**

Here is a point-by-point response to the reviewer **#2** comments and concerns;

**Comments:**
Some decimal places are incorrect in table 1, consider reviewing one by one:
2nd line - Lettuce, 3rd column the 5 should be 63.0;
In the results for Hora Gibe Market, the % for avocado are incorrect 8/15=53.3%;
The same for the total % 13/51=25.5%
The same in the results of Jiren Market

**Response:** The decimal places are thoroughly checked again and errors are corrected in the revised version of the manuscript. Thank you!!

In Table 2 there are also mistakes with two of the decimals.
**Response: Corrected**

The data in table 2 are not uniformly presented. The first three lines describe the parasites identified and the forms found, but the same does not happen for Giardia spp., H. nana, Cryptosporidium spp., Toxocara spp. and Fasciola spp.
**Response:** We fully agree with this comment and to keep the uniformity, only the parasites identified are presented in the revised version of the manuscript.

In Line 315 – authors should write, “Fascioliasis is known to be a neglected zoonosis”

**Response:** Corrected (Line 315). Thank you!!
