# Supplementary material for: Parasitic contamination of fresh vegetables and fruits sold in open-air markets in peri-urban areas of Jimma City, Oromia, Ethiopia: A community-based cross-sectional study
Source: PLoS One. 2024 Mar 21;19(3):e0290655. doi: 10.1371/journal.pone.0290655 (PMC10956833; doi:10.1371/journal.pone.0290655)
Supplement: Attachment — Submitted filename: Response to Reviewers.docx [file pone.0290655.s006.docx]

**Date: December 6, 2023**

Here is a point-by-point response to the staff Editors request;

**Request from Staff Editors:** Before we can proceed, please provide a copy, for our internal records, of the study protocol approved by the Jimma University Institute of Health IRB REF No. IHRPGn/357/21

**Response:** I have attached a copy of the study protocol that has been approved by IRB of Jimma University Institute of Health.
